# Decreased Heart Rate Variability Is Associated with Increased Fatigue Across Different Medical Populations: A Systematic Review

**DOI:** 10.3390/pathophysiology32030046

**Published:** 2025-09-12

**Authors:** Sophie Maria Penfold, James Cunningham, Pauline Whelan, Martin G. McCabe, John Ainsworth

**Affiliations:** 1 School of Health Sciences, Faculty of Biology, Medicine and Health, Division of Informatics, Imaging and Data Sciences, The University of Manchester, Oxford Road, Manchester M13 9PL, UK; james.a.cunningham@manchester.ac.uk (J.C.); pauline.whelan@manchester.ac.uk (P.W.); john.ainsworth@manchester.ac.uk (J.A.); 2 School of Medical Sciences, Faculty of Biology, Medicine and Health, Division of Cancer Sciences, The University of Manchester, Oxford Road, Manchester M13 9PL, UK; martin.mccabe@manchester.ac.uk

**Keywords:** medical populations, medical conditions, heart rate variability, fatigue

## Abstract

Background: Fatigue has been associated with poorer quality of life and increased morbidity in multiple clinical fields. Patients with autonomic dysfunction have been found to experience poorer physiological health, as well as having an increased risk of comorbidity and all-cause mortality. Heart rate variability (HRV) has been documented as a validated tool to assess autonomic function in clinical practice. The aim of this systematic review was to understand the relationship between fatigue and HRV in different medical populations. Methods: A systematic search was conducted in MEDLINE via Web of Science and Scopus. Results: A total of seventeen articles were identified for inclusion. Patients with Chronic Fatigue Syndrome were the most investigated population (*n* = 7), followed by cancer (*n* = 4) and Multiple Sclerosis (*n* = 4). The most implemented fatigue measure was the Multidimension Fatigue Inventory Scale used in four studies and HRV was monitored by electrocardiogram in nine studies. The most recorded and analysed domain for HRV was the frequency parameters. A significant association between increased subjective fatigue and imbalanced metrics of HRV (*p* < 0.05) was identified in fourteen articles. However, results from this review were heterogenous partly owing to the inconsistency with the instruments implemented to monitor HRV and measure fatigue. Additionally, only a small number of medical conditions were investigated, and the patients were predominately older adults (mean age 43.2) and women (64%). Conclusions: Despite these discrepancies, the reviewed evidence suggests that a rise in sympathetic activity and reduced parasympathetic tone are associated with an increased perception of fatigue in medical populations.

## 1. Introduction

Fatigue is a common and complex phenomenon that can be experienced within healthy and medical populations [1]. Research frequently defines fatigue as a subjective, overwhelming and consistent feeling of exhaustion that is not related to prior activity, and which negatively impacts an individual’s ability to function physically and mentally [2,3]. Multiple fields such as sports science, education, occupation and clinical practice have focused on understanding, diagnosing and treating fatigue to better enable an individual’s recovery, rehabilitation and even daily functioning [4]. Consequently, fatigue is understood to be a multifactorial concept that can impact mental and physical performance, as well as cognitive, emotional and motivational responses [1]. The onset of fatigue has been associated with a range of biological mechanisms such as inflammation, alterations to the hypothalamic–pituitary–adrenal axis, immune deficiencies, neuromuscular impairment, circadian rhythm defects, or autonomic nervous system dysfunction, as well as genetic factors, which can also increase the risk of experiencing fatigue [3,5].

Research has identified fatigue as a prevalent and debilitating symptom across a range of medical conditions including, multiple sclerosis, cancer, fibromyalgia, Parkinson’s disease, Chronic Fatigue Syndrome, rheumatoid arthritis, psychiatric disorders, cardiovascular disease, chronic kidney diseases, metabolic diseases, renal diseases and respiratory diseases [6,7,8]. Therefore, understanding and managing fatigue is crucial to improve patient care as experiencing symptoms of fatigue has been associated with reports of negative quality of life and an increased risk of mortality [9].

Fatigue has been commonly reported as a distressing experience pre, during and post cancer treatment and has been considered a late effect, which can negatively impact a person’s life beyond the disease trajectory [5,10]. A systematic review reported the prevalence of fatigue in cancer patients at 49%, whereby ~60% of fatigue frequency occurred in patients with advanced cancer and 62% of incidence rates were recorded pre and during treatment [11]. In addition, patients suffering from chronic illness have been found to have a significantly increased likelihood of burdening the prevalence of severe and chronic fatigue compared to healthy controls [6].

A systematic review by Knoop and colleagues (2021) [9] reported that fatigue was more common in older adults with a prevalence ranging 15–75%; however, these findings were stated to be measurement dependent. Conversely, the severity of fatigue in chronically diseased populations has been found to be associated with younger age though this study stated recall bias may have impacted the reliability of the fatigue measure [6]. Furthermore, studies have found that being female is associated with a higher likelihood of experiencing fatigue [6,9,12]. Lim et al. (2020)’s [12] review and meta-analysis recorded a 0.01–7.62% for fatigue prevalence among chronically fatigued patients and found a 1.5- to 2-fold higher occurrence in women, while the prevalence discovered in children and adolescents was reported at ~0.89%. The type of study design, as well as genetic and environmental factors, were related to influence fatigue incidence [12].

For clinical measures, fatigue is typically assessed subjectively via a questionnaire. Some common types of measures include the Multidimensional Fatigue Inventory (MFI), the Modified Fatigue Impact Scale (MFIS), the Brief Fatigue Inventory Scale (BFI), the Piper Fatigue Scale (PFS), the Fatigue Severity Scale (FSS) and the Chalder Fatigue Scale (CFS) [1,13]. However, there is no universally accepted singular measure to assess fatigue, which means outcomes can be heterogeneous and, therefore, can implicate prognosis and treatment. Furthermore, some measuring scales lack content and construct validity, which jeopardises the reliability of results [13].

Heart rate variability (HRV) is the measured variation between normal-to-normal (NN) sinus beat intervals, which occur between each QRS complex and is typically observed via electrocardiogram (ECG) [14,15]. The NN interval variability can be determined by activity between the parasympathetic nervous system (PNS) and sympathetic nervous system (SNS) [15]. The PNS and SNS work antagonistically to regulate the autonomic nervous system (ANS) to maintain homeostatic balance [16,17]. Consequently, when an internal or external stressor threatens homeostasis, SNS activity is trigged and hormones such as epinephrine and norepinephrine are secreted into the blood stream to incite physiological responses like increasing heart rate and glycogen levels to assist the body with managing the stressor stimuli [16]. Contrariwise, PNS activity physiologically tranquilises the body by reducing heart rate and increasing digestive action thus, restoring balance [16]. In practice, HRV can be analysed at rest or to evaluate reaction to physiological stressors as well as monitor response to disease [18].

Common parameters to monitor HRV include the time and frequency domains [15]. The time domain is measured in milliseconds (ms) and calculations can include the average NN intervals (SDNN), the square root of the average sum of squares between serial NN intervals (RMSSD) or the percentage of difference between succeeding NN intervals that are larger than 50 ms (pNN50), the standard deviation of NN intervals during all 5 min recordings conducted (SDANN), the standard deviation of difference between consecutive NN intervals (SDSD) and the total number of consecutive NN intervals that differ from 50 ms in a recording (NN50 count) [17,19,20]. Research has suggested that ~5–15 min of recorded HRV data is optimal in clinical practice [21]. The frequency domain is typically obtained from ECG data and provides varying amplitudes and frequencies that reflect fluctuations in NN intervals [17]. Analysis of the frequency-based components can be derived from short-term recordings ~2–5 min or over a 24 h period for long-term analysis [20]. The frequency bands can include high frequency (HF), low frequency (LF), very low frequency (VLF), very high frequency (VHF), ultra-low frequency (ULF), total power (TP) and the ratio between LF and HF (LF–HF) [17,19,20]. The standard accepted measures for each band for shorter recording are as follows, HF (0.15–0.4 Hz), LF (0.04–0.15 Hz). For recordings of 24 h, the following band ranges are typically observed, VLF (0.003–0.04 Hz), ULF (<0.003) and TP (<0.4 Hz) [20] HF infers activity regarding vagal tone, whereas LF deciphers sympathetic and parasympathetic tone, as well as baroreflex sensitivity [17,18]. The PNS and SNS balance is understood to be reflected in the LF–HF ratio [21]. The LF–HF ratio assumes that SNS activity drives LF power, whereas PNS tone is responsible for yielding HF power. Consequently, a low LF–HF ratio would indicate parasympathetic supremacy, while a high LF–HF ratio would signify sympathetic tone domination [19].

HRV has been found to decrease from age 15, which coincides with resting heart rate, and female and male parameters have been reported to vary until ~50 years of age [15]. Circadian rhythm can also influence HRV as lower rates are typically observed during waking hours, whereas HRV has been found to increase during sleep [15,16]. Behavioural factors such as BMI, level of physical activity, smoking or drug habits, alcohol abuse as well as environmental factors like climate, medication, or occupation, can influence HRV status [15]. HRV has been used in clinical practice as a diagnostic and prognostic marker to determine ANS dysfunction [22]. Some of the key advantages of using HRV include the ability for measures to be taken in a non-invasive manner that can be cost effective and easily implemented [22]. Recordings of low HRV have been associated with poor health and increased mortality [15,21].

To date, it is unclear whether an association can be determined between fatigue and HRV status in clinical practice. Thus, the purpose of this systematic review is (1) to discover the associations that have been found between fatigue and HRV in medical populations, (2) investigate the methodology used in these studies to determine fatigue and HRV, and (3) understand, when applicable, the differences between fatigue and HRV status in medical populations versus healthy controls.

## 2. Materials and Methods

### 2.1. Literature Search

**Figure 1:**
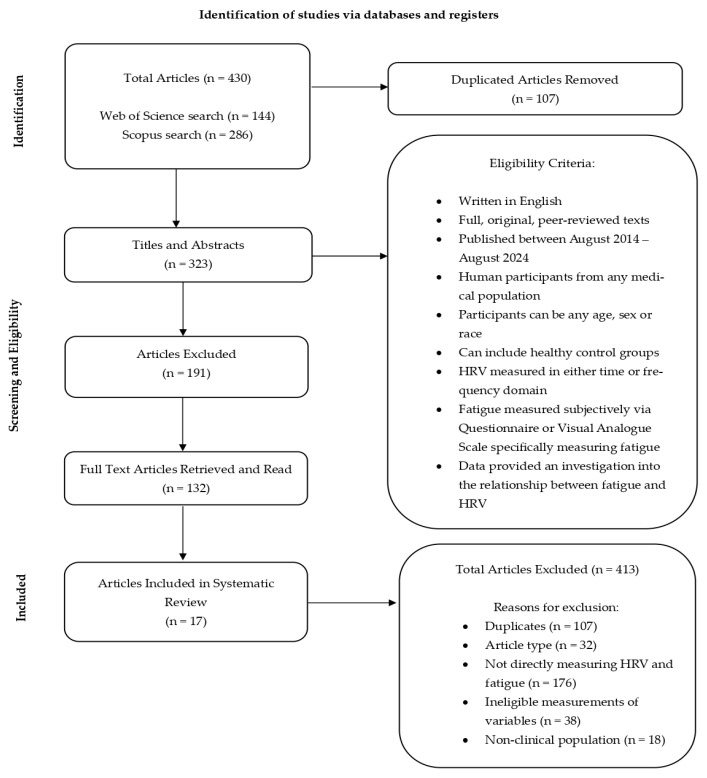
PRISMA flow diagram of article identification, screening and selection for this systematic review.

The systematic review was conducted in accordance with the Preferred Reporting Items for Systematic Reviews and Meta-Analysis (PRISMA) standards (flow diagram, Figure 1) [23]. The databases selected to be systematically searched for this review were MEDLINE via Web of Science and Scopus. To formulate an appropriate search string, the following framework was applied: P—a person with any medical condition, no age limit, any sex/race/ethnicity. I—the measures selected to determine an association between fatigue and HRV. C—studies can be with or without a healthy control group. O—to discover the association found between fatigue and HRV in medical populations. This systematic review was registered with PROSPERO: CRD42024587463.

Searches were conducted on MEDLINE via Web of Science with a combination of MeSH terms and free words for the search terms. MeSH and text terms were expanded and developed following each initial search to ensure a thorough examination of all potentially relevant literature was conducted.

MEDLINE search: “Heart Rate Variability”, or “HRV” and “Fatigu*”, or “Tired*”, or “Exhaust*”, and “Patient*”, or “Service User*”, and “Diseas*”, or “Sic*”, or “Conditio*”, or “Medica*”, or “Ill*”, or “Clinica*”.

Another search was completed on Scopus using the same search string terms as MEDLINE. The search on Scopus was additionally restricted to papers that were written in English and journal articles. Articles were also required to have been published between 7 August 2014 and 7 August 2024, which was the date the database search began. The final database search was conducted on 11 December 2024.

### 2.2. Inclusion and Exclusion Criteria

Studies were included based on the following inclusion criteria: (1) written in English; (2) human participants; (3) studies published in the last decade; (4) full, original, peer-reviewed journal articles; (5) patients with any medical condition; (6) studies can include a healthy control group; (7) no age, sex, race, ethnicity restrictions; (8) HRV must have been measured objectively in either the time or frequency domains and the following calculations were accepted: SDNN, SDANN, SDSD, RMSSD, NN50 count, pNN50 or LF, HF, VLF, VHF, ULF, TP or LF–HF; (9) fatigue can include physical or mental measures, which are subjectively given via a questionnaire or a Visual Scale; (10) data must provide an association between fatigue and HRV without any other variables.

The ineligibility of studies was based on the following exclusion criteria: (1) HRV measured non-linear or via subjective questionnaires; (2) fatigue measured via a questionnaire or Visual Analogue Scale that do not specifically measure fatigue but instead multiple symptoms; (3) measure of fatigue or HRV determined via a task; (4) study designs such as case studies, trial protocols, conference papers, book chapters, letters, thesis, or review articles; (5) results that include cofounding variables alongside fatigue and HRV analysis.

### 2.3. Researchers

The articles were independently searched by two reviews (S.M.P.) and (J.C.). The title and abstracts were read by each reviewer and potential articles for inclusion were downloaded and the text was read to determine eligibility. To minimise the risk of bias, the reviewers were blinded to one another’s article selection. The reviewers met and presented one another with the articles they felt were eligible for the systematic review based on the inclusion and exclusion criteria. Any disagreement regarding article selection was settled during a formal discussion. The researchers agreed to include 17 articles in this systematic review.

### 2.4. Quality Assessment

To assess the quality of each of the studies methodology, the lead researcher used the Mixed-Method Appraisal Tool [24] to critically evaluate the different quantitative study designs that have been included in this review. Most articles were awarded a ‘yes’ response to each of the questions that evaluated the articles methodology with additional comments for evidence added to support the decision made.

## 3. Results

### 3.1. Study Process

The initial searches identified a total of 430 articles, whereby 107 were duplicates and thus removed. The titles and abstracts of the remaining 323 articles were screened for eligibility based on the inclusion criteria and of these, 191 articles were deemed ineligible. A total of 132 full texts were downloaded and read by the researchers. The researchers agreed to exclude 115 articles. Overall, 306 articles were excluded as 32 were ineligible article types, 176 did not directly measure a relationship between fatigue and HRV, 38 articles implemented HRV or fatigue measures that did not meet the inclusion criteria, 18 of the articles studied non-clinical populations and 42 articles did not meet the study design criteria for inclusion. Therefore, 17 articles were finally selected to be narratively synthesised for the purpose of this systematic review.

### 3.2. Characteristics of Articles Included

Table 1 displays the characteristics of each study included in this systematic review.

### 3.3. Participant Population

The clinical populations investigated included cancer 24%, (*n* = 4 articles), Chronic Fatigue Syndrome (41%, *n* = 7), multiple sclerosis (24%, *n* = 4), systemic lupus erythematosus (6%, *n* = 1), chronic orthostatic intolerance (6%, *n* = 1) and Spleen-Qi deficiency syndrome (6%, *n* = 1). A healthy control group was included in nine of the articles. The total number of patients was 1, 135 and the total number of healthy-matched controls was 296. The mean age for 16 studies was 43.2 for the medical population and 39.7 for healthy controls, one study did not report the mean age of patients but instead stated the ages ranged from >30 to <50 years. The medical cohort was comprised of 729 women and 406 men, whereas the controls had 162 women, 132 men and 2 nonbinary participants.

The studies had similar exclusion criteria, which included no history of cardiovascular disease, any cardiac arrhythmias, a psychiatric disorder, an infection, inflammatory disease, a severe respiratory disease, diabetes, pregnancy, breast feeding, a history of alcohol or substance abuse, anaemia, hypertension, a thyroid-related disease, sleep apnoea or taking hormonal medication. Pre-assessment requirements included overnight fasting, refraining from alcohol, caffeine or smoking 3–12 h prior to HRV assessment as well as no physical activity permitted 24 h before testing. The time of assessments varied, but all testing occurred between 8 a.m. and 6 p.m. A significant association was found between fatigue and HRV in 14 of the articles (*p* < 0.05) that are presented in Table 1.

### 3.4. Cancer

Four of the articles reviewed investigated the association between fatigue and HRV in patients with cancer. One study was cross-sectional [30], the next was a comparative study [35], there was also a controlled study [27], and the final study was a randomised control design [41]. The cancer types of patients investigated included lung, breast, thyroid, gastrointestinal, nasopharyngeal carcinoma, hematologic and brain tumour. All the participants were recruited from hospitals and each of these articles defined fatigue in relation to cancer-related fatigue (CRF), which was stated as a multifactorial phenomenon that was related to cancer and cancer treatment and remained persistent, long-lasting and, therefore, negatively impaired the individual’s daily functioning and quality of life. There were five different subjective psychometric scales used to measure fatigue. One study examined fatigue using the Multidimensional Fatigue Symptom Inventory, which has 20 items to assess fatigue on a 5-point Likert scale across general, physical, mental dimensions as well as evaluating motivation and activity levels. The questionnaire has good reliability with Cronbach’s α = 0.84 [30]. Two of the studies instigated the Multidimensional Fatigue Symptom Inventory-Short Form (MFSI-SF), which has 30 items scored from 0–4 and accesses general, emotional, mental and physical fatigue as well as Vigor. The Cronbach’s α = 0.90; the α coefficient of internal consistency ranges 0.83–0.84 [41]. Other measurements used was the Fatigue Severity Scale (FSS), which consists of 9 statements to access magnitudes of physical and mental fatigue using a 7-point Likert scale. The scale has acceptable reliability with the Cronbach’s α = 0.92 [35]. One study also used the Brief Fatigue Inventory (BFI) scale, which has 9 items that evaluate fatigue on a 10-point Likert scale (Cronbach’s α = 0.88) [35]. The BFI accesses mental, physical and cognitive aspects of fatigue. The final measure was the German version of the Functional Assessment of Chronic Illness-Fatigue questionnaire (FACIT-F) (Cronbach’s α = 0.95–0.96) [27,42]. The FACIT-F measures fatigue in relation to daily activities and functioning.

To measure HRV, two of the studies gathered data via ECG reading, whereby one of these studies recorded frequency domain metrics for 5 min [41] and the other collected both time and frequency measures for 8 min [35]. Two of the studies used ambulatory devices to assess HRV. One study used a three-electrode 24 h Holter monitor, which gathered frequency and time domain parameters [30]. The other study used a LifeShirt System which collected respiratory sinus arrhythmia (RSA), which is a measure of HF variability, during a laboratory procedure and then continued to record HRV for 24 h during the patient’s daily routine [27]. Two of the articles reported no significant difference between fatigue and HRV in cancer patients. There was no significant correlation reported in patients undergoing craniotomy resection (r ≤ 0.16, *p* ≥ 0.25) [30]. The other study, which investigated patients with various cancer types suffering from CRF, stated that it did not determine any statistical significance between fatigue and HRV, but the researchers did not report the results [35]. The absent relationship between fatigue and HRV in these studies may be related to the age of participants as all were older adults with the mean age across studies at 53 years; thus, other physiological mechanisms could be influencing ANS function. The studies also reported that factors related to the recovery and daily routine [30], as well as the medication patients were receiving [35] had been uncontrolled for. Moreover, one study was performed in a clinical environment and involved a short recording of HRV which may not reflect the true fluctuation of HRV that could be observed if monitoring occurred longer in a remote environment that considered both day and nighttime reading [35].

However, two of the studies discovered a statistical significance between fatigue and HRV in cancer patients. The first study to review investigated patients with nasopharyngeal carcinoma who began Tai Chi for 60 min, 5 times per week during chemoradiotherapy versus a patient control group who received usual care [41]. Evaluation of CRF was determined by patients MFSI-SF score. To account for the 31 participants who dropped out during this study, analysis was conducted for intention-to-treat (114 participants) and for pre-protocol (83 participants). Participants in this study were assessed pre (T0) and post (T1) chemotherapy. Pre-protocol analysis before and after chemotherapy revealed a significant correlation coefficient between CRF and normalised LF–HF ratio (nLF-nHF) (0.767 and 0.761, *p* < 0.01). Furthermore, when age and sex were controlled, the adjusted coefficients were 0.728 and 0.732, *p* < 0.01. The intention-to-treat analysis correlation coefficient was 0.752 and 0.798, *p* < 0.01 and when adjusted for age and sex, remained significant 0.725 and 0.779, *p* < 0.01 [41]. The researchers observed a higher increase in nLF-nHF ratio after chemotherapy suggesting that patients had higher sympathetic tone and, therefore, the ANS was unbalanced, which could explain the higher reports of fatigue.

A study evaluated the autonomic function of survivors from allogenic hematopoietic stem cell transplantation in remission and matched healthy controls [27]. Patients were sub-grouped into high (HFP), medium (MFP), non-fatigued (NFP) based on FACIT-F scores and controls (CTL). Participants were then requested to complete a submaximal fitness test on a bicycle ergometer. After 20 min rest, participants were fitted with an ambulatory LifeShirt, which monitored RSA during a laboratory procedure that involved tasks to stimulate daily activities (e.g., sitting and speaking). Participants were then instructed to leave and continue their daily lives wearing the LifeShirt for an additional 24 h. During patients’ and controls daily lives, a general linear model found RSA to be significant with fatigue scores (F = 6.57, *p* < 0.001), which remained significant when controlled for patients’ and controls fitness scores (F = 3.44, *p* = 0.002). In addition, contrast analysis found a significant difference between HFP vs. NFP (t = −2.39, *p* = 0.02, d = 0.72). There was no significant difference found for MFP vs. NFP (*p* = 0.08) or CTL vs. NFP (*p* = 0.27). When controlling for fitness scores, patients who had clinically higher fatigue had increased significant results compared to those who were classified as non-fatigued (t = −2.74, *p* = 0.007, d = 0.72). A similar trend was also observed in those with medium fatigue when compared to non-fatigued patients (t = −1.89, *p* = 0.06, d = 0.51) but this was not significant. There were no significant differences found for controls when compared to non-fatigued patients (*p* = 0.62). This study demonstrates that CRF can relate to a reduction in RSA values, which reflect cardiac vagal tone. Moreover, patients with higher fatigue were found to have reduced RSA in comparison to medium and non-fatigued patients. The RSA reflects the changes in the HF component that are interrelated to the respiratory cycle [43,44]; thus, the results from this article support that reduced vagal tone can be associated to an increased perception of fatigue.

### 3.5. Multiple Sclerosis (MS)

There were four studies that evaluated the relationship between fatigue and HRV in patients with MS. One study was a clinical trial, which investigated the association between HRV and subjective trait and time on task (tot) fatigue prior and after completing an acoustic vigilant test [38]. In this study, fatigue was considered in two forms: trait fatigue, which is a chronic occurrence that effects mental construction, and tot whereby fatigue is amplified by external factors. Participants were recruited from a rehabilitation centre in Germany and were required to complete the 9-item Fatigue Severity Scale (FSS) and the 20-item Fatigue Scale for Motor and Cognition (FSMC) to assess trait fatigue, as well as giving a score on a Visual Analogue Scale (VAS) to measure tot fatigue. The FSS measures physical and mental fatigue, which is like the FSMC domains that access the physical and cognitive impact of fatigue. HRV was monitored throughout the procedure via a Blood Volume Pulse Sensor, which collected both time and frequency parameters. In this study, age, duration of disease and symptoms, disability status, MS type and depression were controlled for. The results found the cognitive fatigue score from the FSMC were able to be significantly predicted from VLF and HF values (R^2^ = 0.218, F = 4.558, *p* = 0.007). However, HRV parameters was not a significant predictor for FSMC or FSS scores for motor fatigue. These findings suggest that MS patients who experience an increase in parasympathetic activity coupled with a decrease in sympathetic tone experience a higher perception of cognitive fatigue.

In addition, forward regression analysis found that tot fatigue was a significant predictor by SDNN (Beta = −0.793, t = 3.136, *p* = 0.003) and pNN50 (Beta = 0.994, t = 3.934, *p* ≤ 0.001). The time domain results suggest that subjective measures of tot fatigue were experienced when ANS was imbalanced due to an increase in parasympathetic tone that was induced by the external factors of the task. However, SDNN is representative of PNS and SNS modulation, suggesting a decrease in sympathetic tone can also relate to tot related fatigue in MS. Despite these findings, the authors found no significant correlation between the HRV parameters and scores relating to motor fatigue. Though the article suggests that cognitive fatigue in MS may relate to an imbalance in ANS function, the evidence does not support a connection between motor fatigue and HRV. Therefore, the extent of which autonomic dysfunction can have on domains relating to physical fatigue, should be considered with caution. Nevertheless, the evidence from this study supports that patients with MS performance can be less responsive due to the association between heightened parasympathetic tone and increased cognitive fatigue.

A randomised control trial recruited 50 MS patients from a Median Clinic to access how two forms of biofeedback treatment could affect subjective fatigue scores and provoked fatigue performance [39]. The intervention groups were self-alert training (SAT) or progressive muscle relaxation (PMR) and the procedure lasted two days. Fatigue was understood as a symptom of MS that related to automimic dysfunction. The study used multiple fatigue measurements including the VAS, FSMC and FSS to assess subjective fatigue, whereas a vigilance task was used to deceiver induced fatigue. Based on FSMC median scores, patients in each intervention group were sub-grouped to either weak–moderate fatigue or severe fatigue. Physiological measures were collected via Blood Volume Pulse Sensor NeXus-4 Biofeedback-System and software analysed HRV in the time domains SDNN and pNN50. There was a significant three-way interaction effect on day, sub-group and fatigue scores with SDNN F = (1,46) 5.051, *p* = 0.029 and pNN50 F = (1,46) 7.230, *p* = 0.010. MS patients grouped with severe fatigue in both the SAT and PMR group had lower mean values of SDNN and pNN50 compared to those classified with moderate-weak fatigue. Furthermore, dependent *t*-test post hoc analysis revealed PMR patients classified as weak–moderate fatigued had a significant rise in SDNN (*p* = 0.001) and pNN50 (*p* = 0.008) post intervention. However, there was no significant findings for those classified with severe fatigue in the PMR group or for any patients in SAT group. These results found that patients with moderate-weak fatigue experienced an increase in vagal tone, whereas those who experienced higher fatigue did not display any significant improvement to ANS function after the intervention. These findings support that medical cohorts with reduced HRV subjectively suffer from a worsening sense of fatigue.

A cross-sectional study evaluated cardiovascular autonomic dysfunction in MS patients during a head-up tilt test (HUTT) [37]. The study did not clarify where MS patients were recruited from. In addition, this study did include a healthy control group; however, the relationship between fatigue and HRV was only investigated in MS patients. In this article, fatigue was considered as a disabling experience that related to cardiovascular autonomic dysfunction that was specific to MS. The patients were divided into two groups: progressive MS and relapse–remitting MS. The patients were requested to complete the Chalder Fatigue Scale, which is an 11-item questionnaire that assess mental and physical fatigue and has been reported to have good reliability in studies with MS (Cronbach’s α = 0.89) [13]. The Task Force Monitoring System two-channel ECG was attached to participants to monitor the absolute and normalised values of frequency for HRV. The results found that physical fatigue scores predicted the balance of the LF–HF-RRI ratio (HRV ratio between the low- and high-frequency bands) (β = 0.338, SE = 0.05, t = −2.672, *p* = 0.010). The findings suggest that enhanced sympathetic activity can cause an ANS imbalance, which consequently increases physical fatigue. However, there was no significant relationship between any of the other frequency domains with physical fatigue, nor was mental fatigue scores associated with any of the monitored parameters. Together, the three articles reviewed suggest that fatigue in MS patients can be predicted by ANS dysfunction; however, the physical and mental experiences can vary and may also depend on other physiological and external factors.

The final study to review was conducted by Gashi and colleagues [29] who examined the feasibility and reliability of a wearable device dataset to determine if remote objective and subjective measures could be used in clinical settings to monitor patients with MS. The observational study investigated 55 patients with MS and 34 healthy controls who were all recruited from a University Hospital. Participants were involved in the study for 14-days and were given an Eversion Armband wearable device whereby continuous monitoring of HRV was recorded via PPG in both the time and frequency domains. Fatigue was determined pre and post the 14-day period for MS patients via the FSMC questionnaire, and all participants were asked to record their perceived fatigue three times a day via the VAS 1–10 scale, which was issued via the Querium application on their smartphones. For the fatigue and HRV parameter correlations, only MS patients were investigated by using the mean score provided by the two FSMC assessments. The controls were assigned a FSMC score of 0. A negative significant correlation was discovered between fatigue and some HRV parameters (pNN50 r = −0.40, *p* = 0.001, NN50 r = −0.41, *p* = 0.01, SD2 r = −0.47, *p* = 0.001, LF r = −0.26, *p* = 0.001) and a positive correlation was found between LF–HF ratio and fatigue scores (LF–HF r = 0.26, *p* = 0.001). However, the RMSSD, SD1 and HF parameters were not significant with FSMC scores. Overall, HRV parameters were reduced in patients with higher FSMC fatigue scores. The findings suggest that MS patients who experience higher symptoms of subjective fatigue have reduced parasympathetic tone, increased sympathetic activity and vagal imbalance. This study was conducted in patients free-living environment; therefore, variables relating to daily and weekly behaviour, e.g., sleep time or physical activity, could have affected results and may explain the discrepancies observed between HRV parameters and increased fatigue. However, the study provides evidence of success for using an objective assessment of HRV, which is associated with subjective measures of fatigue in a clinical cohort. This study was also one of the only studies in this review to observe the association between fatigue and HRV whilst patients are in a remote environment [29].

### 3.6. Chronic Fatigue Syndrome (CFS)

Seven of the articles in this review evaluated the association between fatigue and HRV in individuals suffering from CFS. One article previously discussed compared CRF patients against patients with CFS [35]. Like CRF, CFS was defined as a continuous and intensifying experience that effects the physical functioning of an individual. Though no significant relationship was discovered between fatigue and HRV in CRF group, there was a positive correlation between scores from the Fatigue Severity Scale (FSS) and LF power (r = 0.484, b = 0.047, *p* = 0.036), which suggests increased sympathetic tone. Moreover, a negative correlation was also determined for FSS scores and HF power (r = −0.508, b = −0.051, *p* = 0.026) and RMSSD (r = −0.509, b = −0.032, *p* = 0.026). As both of these parameters are understood to relate to parasympathetic activity, the results from this research suggest that CFS patients experience higher bouts of fatigue from ANS dysfunction due to a reduction in parasympathetic activity that is coupled with a surge in sympathetic tone [35].

Two of the articles were cross-sectional case–control studies that followed on from one another to assess fatigue and HRV in CFS women [28] and then CFS men [26] against healthy-matched controls. Fatigue was understood as a multidimensional concept that negatively altered an individual’s physical and mental functioning and was unable to be resolved from rest. Both studies conducted the same procedure. Patients were recruited from a local hospitals outpatient unit and controls were enrolled from the community via posters and word of mouth. The Fatigue Impact Scale (FIS-40) had 40 items, which accessed the physical, cognitive and psychosocial dimensions of fatigue on a 0 (no fatigue) to 4 (severe) scoring system. A global fatigue score can then be calculated on completion. The Cronbach’s α ≥ 0.87 insinuates good consistency [45]. HRV was measured in three five minute blocks by a Polar Electro H7 chest strap that was synced via Bluetooth to a smartphone app that allowed the FitLab System to analyse time and frequency parameters.

The first study that investigated CFS women and controls found a significant negative association between all FIS-40 scores and all time HRV domains, as well as HF and HFnu (*p* < 0.01) [28]. There was a positive association between all FIS-40 scores and LF–HF ratio values (*p* < 0.05). The LF parameter was only significant with physical and cognitive FIS-40 domains (*p* < 0.01). The results indicate that CFS and HC women with a reduced interval times between RR intervals can lead to a lower HRV and thus can experience higher symptoms of fatigue. In reference to findings regarding the higher LF–HF ratio, the increase in LF activity and reduction in HF suggests the increase in sympathetic tone is linked to the experience of fatigue. Furthermore, a simple regression analysis found a significant relationship between the mean RR (*p* = 0.0050), HFnu (*p* = 0.0067) and RMSSD (*p* = 0.0268) with higher global FIS-40 scores in CFS women but no significant findings were detected in healthy controls. These results further support that lower HRV can increase the experience of fatigue within medical populations compared to healthy controls.

As a follow-up study, researchers investigated the relationship between fatigue and HRV in male patients suffering CFS against healthy-matched controls [26]. In this study, a significant negative association was discovered between the physical FIS-40 scores and pNN50 (r = −0.279, *p* = 0.049) when evaluating all participants. The other HRV values were not significant with the remaining fatigue scores. However, a simple regression analysis found men suffering CFS physical FIS-40 scores had a significant negative relationship with SDNN (β = −0.487, *p* = 0.0047), RMSSD (β = −0.394, *p* = 0.0258), pNN50 (β = −0.378, *p* = 0.0033), LF (β = −0.537, *p* = 0.0015) and HF (β = −0.421, *p* = 0.0165). There was no significant association discovered for healthy control. The findings suggest that reduced HRV increases physical fatigue in men with CFS. However, the negative results for LF contradict the previous findings as this would suggest in men with CFS, a reduction in sympathetic tone can cause physical fatigue. A plausible explanation put forward by the researchers was that LF can also be influenced by vagal tone, which could explain why this value was reduced in these participants [26,46]. The results from these two studies supports that a reduction in HRV can increase the subjective experience of fatigue; however, the extent of which dimensions of fatigue can be affected varies between women and men [26,28].

The next study to investigate CFS population was a cross-sectional study that recruited a women cohort involving 17 patients and 17 healthy controls to investigate cerebral blood flow and assess the relationship between fatigue and HRV [25]. In this study, fatigue was defined as a multidimensional phenomenon, which persisted for over six months and was recognised as highly prevalent and costly in the US. Fatigue scores were collected via VAS 0-100 scale, which ranges from ‘no fatigue’ (0) to ‘the most intense fatigue’ (100) and the internal consistency has been reported at 0.96 and 0.91 [47]. HRV was monitored by an ECG recording for 15 min whilst participants were in supine resting. Both time and frequency indices were measured and analysed. A significant negative correlation was found between rating of fatigue and total HRV power in CFS patients (r = 0.70, *p* = 0.02). The results stated that a lower total HRV power value related to a higher report of subjective fatigue. As the total HRV power represents the collective variability of all frequency domains, the findings suggest that the sympathetic and parasympathetic function is altered and reduced in individuals suffering intense symptoms of fatigue. However, the study did not discover any other significant relationships between fatigue scores and remaining HRV values. Moreover, researchers declared that the significant finding was only produced when an outlier was removed from the initial analysis.

A pilot study investigated the longitudinal effects of daily seated isometric yoga on autonomic functions in patients with CFS [33]. For this study, analysis was conducted on data retrieved from a previous RCT the authors had conducted [48]. There were 30 CFS patients recruited from a hospital outpatient unit, and these participants were allocated to either the yoga or ‘treatment as normal’ condition. Due to issues with discomfort, the control group’s ANS function was not assessed; therefore, only the patients in the yoga group had fatigue and HRV data correlated. Physiological and subjective measures were assessed pre and post the 2-month intervention in the afternoon, whereby HRV was recorded for 2 min via ECG to monitor parameters in the frequency domain, and fatigue was assessed by scores from the Chalder Fatigue Scale. There was a positive correlation discovered between HF and Chalder Fatigue scores (r = 0.705, *p* = 0.042). None of the other HRV parameters showed significance with fatigue. The results from this study suggest that fatigue scores were lower in CFS patients who displayed reduced vagal tone [33]. In this study, the CFS patients who participated in yoga were found to have lower HF during daytime assessment. Though an association was discovered, the findings between fatigue and HRV are inconsistent with other literature in this review. A plausible explanation for the reduction in parasympathetic tone post-intervention suggested by the authors was that the CFS patients had been sedentary prior to the yoga practice and due to the intervention, the increase in physical activity could be part responsible for reduced vagal activity during the day [33].

Another study investigated ANS function at rest and then during different breathing states in patients with CFS (*n* = 34) against patients with post-COVID-19 condition (PPC) (*n* = 29) [36]. The study also included 32 healthy-matched controls. HRV response to deep breathing was assessed via ECG in three parts: during 5 min of spontaneous breathing, controlled breathing at 12 breathes per minute for 2 min and finally controlled breathing at 6 breathes per minute for 2 min. The frequency domains monitored included, TP, HF, LF and VLF. Perception of fatigue was determined by scores from the Multidimensional Fatigue Inventory (MFI) scale. No correlations were discovered between any HRV parameters and fatigue for the PPC group or the healthy controls. In patients with CFS, there was a negative correlation between the general fatigue domain scores of the MFI and HF (r = −0.522), and TP (r = −0.444) during the 12 breaths per minute assessment; however, neither was significant (*p* > 0.01). Although the results from this study suggest that ANS dysfunctional can be associated with increased general fatigue in patients with CFS, the findings were not consistent for all patients within this study. The correlation was only observed for TP and HF and, therefore, was not constant across the other frequency parameters of HRV. Furthermore, TP and HF was only correlated with scores relating to general fatigue, and not with any of the remaining sub-scales of the MFI. In addition, an association was only found for patients with CFS but not in patients with PPC. The findings in this study support the complexity of assessing fatigue via HRV in different medical cohorts [36]. However, the results provide further evidence that ANS dysfunction is observed more often in clinical populations than healthy controls.

The final article to evaluate fatigue and HRV in individuals suffering from CFS was a cross-sectional study conducted on adolescents aged between 12 and 20 years 6 months after Acute Epstein–Barr Virus (EBV) infection diagnosis [31]. The adolescents were grouped as those with chronic fatigue 6 months post-EBV, (EBV CF+), non-chronic fatigued 6 months post-EBV (EBV CF−) and healthy controls. Patients were recruited from a hospital, and the controls were predominately friends of the patients who attended the follow-up. CFS was stated to reduce an adolescent’s quality of life and negatively impact their psychosocial development, relationships and academic prospects as well becoming a costly burden in society to treat and manage. The study assessed fatigue via the Chalder Fatigue Questionnaire (CFQ). For HRV, participants were connected to a Task Force Monitor and the ECG recorded data 5 min during supine rest, 5 min of controlled breathing via beep signalling and 3 min of standing. The researchers reported a weak negative correlation between LF–HF ratio during controlled breathing and total CFQ score (τ = −0.21, *p* = 0.005) in EBV CF+ adolescents only. No other significant relationships were discovered between fatigue and HRV in this study.

### 3.7. Other Medical Populations

There were three articles that investigated additional medical populations. One study was cross-sectional and included a 12-week aerobic exercise intervention to assess the relationship between HRV, markers of stress and oxidation as well as patient reported outcomes in persons diagnosed with Systemic Lupus Erythematosus (SLE) [32]. The study included 55 women who were recruited from the disease unit within a hospital. Fatigued was scored using the Multidimensional Fatigue Inventory (MFI) questionnaire and HRV was measured using a Polar V800 chest strap for 10 min after a 5 min resting period in supine to derive time and frequency values. The relationship between HRV and fatigue was only assessed prior to the intervention on all women. The only significant result reported was that MFI physical scores positively correlated to LF–HF ratio (r = 0.30, *p* ≤ 0.05). In addition, significant findings were further analysed by quantile regression model, which discovered that the LF–HF ratio is associated with MFI physical fatigue scores (unstandardised coefficient B = 0.89, 95% CI 0.15–1.62, *p* = 0.019). As the LF–HF ratio is understood to represent sympathetic and vagal balance, results from this study indicate that a rise in sympathetic tone can increase symptoms of physical fatigue within this patient cohort.

Another cross-sectional pilot study recruited patients with Spleen-Qi Deficiency Syndrome (SDS) and healthy volunteers from a hospital university to investigate HRV and the effects on fatigue [34]. The study did not specifically define fatigue but referred to it as a symptom of the disease. The Fatigue Impact Scale-Spanish version (FIS) included 10 items to assess physical, mental and psychosocial fatigue across a 5-point Likert scale with reported Cronbach’s α = 0.81–0.9728. HRV was recorded for 5 min following a 10 min supine resting period and analysis was conducted in time and frequency domains. There was a linear, positive correlation between FIS scores and LF–HF ratio of patients (r = 0.48, *p* < 0.01) and FIS scores were also found to have a negative correlation with HF index power (r = −0.37, *p* < 0.01). These results provide further support that symptoms of fatigue are scored higher in patients with increased sympathetic activity and reduced parasympathetic tone.

The final article was a retrospective study that recruited patients from clinic with Postural Tachycardia Syndrome (POTS), a common form of chronic orthostatic intolerance (COI) [40]. The study aimed to investigate the association between various physiological factors and orthostatic response as well as the severity of symptoms including fatigue during the HUTT. This study did not state a definition for fatigue but referred to it as a symptom of COI. To measure fatigue, the researchers used the 9-item Fatigue Severity Scale (FSS), which scores fatigue in relation to how it effects physical and social interaction. HRV was recorded by an ECG for 5 min at baseline then at minute 1 and 10 during the HUTT. A multiple linear regression final model revealed a significant association between absolute power of LF (LF-HRV) during the HUTT and higher FSS scores (t = −2.719, *p* = 0.008). In this study, the researchers concluded that the reduction in LF-HRV reflected the arterial baroreflex function. This study suggests that LF-HRV reflects the maintenance of baroreflex whereby a change in blood pressure can cause heart rate to increase or decrease, respectively, and therefore impact symptoms of fatigue in POTS patients. Other research has found that LF can be a measure of baroreflex activity rather than sympathetic tone [49].

## 4. Discussion

This is the first systematic review to investigate the relationship between fatigue and HRV across different medical populations. In this review, 14 of the articles identified a significant relationship between fatigue and HRV (*p* < 0.05). Overall, the findings suggest that patients with increased SNS activity and reduced PNS tone are more likely to score higher on subjective fatigue scales. Furthermore, the articles in this review give evidence that HRV can be measured objectively to monitor fatigue in clinical practice. The evidence in this review provides further support that biological mechanisms can be impacted by the pathology of disease. Other literature has stated that within multiple medical conditions, the impact of a disease on immune cell function, inflammation markers, central nervous system, neuroendocrine system, ANS, hypothalamic–pituitary–adrenal axis or circadian rhythm can impair physiological function, and thus increase the likelihood of experiencing fatigue. It is plausible that ANS dysregulation could be caused by a surge in inflammatory activity, which heightens sympathetic response, and thus decreases heart rate variability and increases fatigue [3,50]. Moreover, a lower HRV coupled with increased fatigue across the different medical conditions resulted in a poorer subjective ability to function physically, mentally and cognitively within the cohorts investigated. These findings support the need to improve the physiological understanding associated with fatigue to assist in appropriate management of symptoms to improve quality of life during and beyond the disease trajectory. However, the results from the articles reviewed are inconsistent and thus are to be considered with caution as the HRV metrics that were utilised differ across each medical condition. Furthermore, the fatigue domain, which was affected by various HRV parameters was also not constant. Therefore, the findings are heterogenous within and across the medical populations investigated. Nevertheless, 14 studies provided empirical evidence, which support an association between fatigue and HRV in medical cohorts.

Each study reviewed had a similar definition for fatigue and understood it to be a complex, multidimensional phenomena that is consistently experienced for over 6 months, is physically and mentally debilitating, and therefore has a negative effect on the persons quality of life. Compared to patients, there were no significant associations found between higher fatigue scores and reduced HRV in the articles that evaluated healthy controls. These findings can be strengthened by existing research, which has discovered that in comparison to healthy-matched cohorts, clinical populations experience autonomic dysfunction [18,51,52]. A meta-analysis investigating HRV in patients with CFS, irritable bowel syndrome and fibromyalgia found a significant small–medium association in reduced RMSSD and a significant medium-large association in decreased HF power in all patients but not in healthy-match controls [53]. A reduction in parasympathetic tone has been associated with autonomic dysfunction in clinical populations, which has been associated with adverse physiological effects [53,54]. A review by Garis and colleagues [44] documented patients with MS suffer from ANS dysfunction, which relates to fatigue and is more probable in patients versus healthy controls. In addition, a systematic review investigating ANS function in breast cancer survivors, found that lower HRV was more prevalent in survivors compared to healthy controls [54]. Research has concluded that longer rates of survival can be associated with higher HRV [55,56]. Therefore, increasing HRV could improve health-related outcomes, such as fatigue as well as reduce risk of comorbidities and mortality [53].

There were nine articles that applied correlations to establish the relationship between fatigue and HRV. Two studies discovered a strong association between increased fatigue and reduced HRV [25,41], there were two studies that established a moderate relationship [34,35], whereas six studies reported a weak relationship [26,28,29,31,32,34], and one very weak [28]. The results suggest that heightened sympathetic nervous system activity and reduced parasympathetic tone can increase subjective domains related to physical, emotional, cognitive and mental fatigue. The findings in this review can be supported by earlier studies that have discovered a relationship between increased fatigue and lower HRV in cancer survivors [57,58]. Crosswell et al. [57] found that young breast cancer survivors with reduced RMSSD experienced increased fatigue. In addition, Fagundes and colleagues [58] investigation into fatigue and ANS response in breast cancer survivors found that lower values of RMSSD and HF were associated with higher ratings of subjective fatigue. These results support the findings in this review which conclude reduced parasympathetic tone and increased sympathetic activity are associated with a higher perception of fatigue.

Three of the studies in this review were trials that included a physical activity intervention. One study evaluated fatigue and HRV in patients prior to a 12-week block of aerobic exercise on patients with SLE [32], another study investigated the effects of a 12-week Tai Chi program on cancer patients [41] and the final intervention investigated the effects of 2 months of daily isometric yoga practice on ANS function and fatigue in CFS patients [33]. All three studies found a significant association between fatigue and HRV. Two of the studies did not directly evaluate the association between fatigue and HRV post-intervention; thus, it is unknown if the intervention improved the relationship between fatigue and HRV in those patients [32,41]. The previous literature can provide empirical support that physical activity can improve HRV and lower fatigue in medical and healthy populations [59,60,61]. In contrast to these findings, one study in this review found 2 months of isometric yoga practice to reduce fatigue in patients with CFS; however, the reduction in fatigue was correlated to reduced vagal tone [33]. The lowered parasympathetic activity may be related to the time of ECG recording as HF band has been reported lower during the daytime in patients with CFS [62]. The conflicting evidence between these studies further supports the need for research to be conducted longitudinally. Additional research could assist in understanding if fatigue can be associated with HRV when monitored at alternative time points. In addition, if physical activity is successful at reducing fatigue and improving HRV, this could be an intervention medical professionals could use to help manage fatigue.

The articles in this review had more women than men in both the medical population (729 women versus 406 men) and the healthy controls (162 versus 132, respectively). Empirical evidence has found that factors such as sex and age, as well as BMI, can significantly impact HRV and fatigue [52,63,64]. The literature has documented that women have reduced LF and SDNN values compared to men, whereas HF is typically higher in women [18]. Ying-Chih and colleagues [52] conducted a systematic review and meta-analysis whereby sub-group analysis discovered lower HRV in women compared to men. In addition, studies have discovered women experience more fatigue symptoms compared to male counterparts [64]. There were two studies in this review that presented differentiating results from women and men [26,28]. An increased perception of fatigue was associated across multiple domains related to physical, cognitive and psychosocial fatigue with HRV parameters in both the time and frequency indices for women. However, only the FIS-40 domain relating to physical fatigue was significantly associated with time and frequency indices in male patients [26,28].

In addition, the mean age of the studies included in this review was 42.3; thus, the association between fatigue and HRV in younger clinical cohorts remains under-investigated. HRV has been found to reduce ~15% every 10 years in both time and frequency parameters. However, the effect of sex on HRV has also been found to diminish as individuals age [18]. Previous research has found sex-related HRV differences are greater in younger adults [63]. Therefore, more research is necessary to understand the impact age could have on the relationship between fatigue and HRV.

### 4.1. Limitations

There are some limitations within this review. Firstly, the articles reviewed cannot be generalised to all medical cohorts as they are predominately limited to cancer, MS and CFS. In addition, there were four studies that investigated other medical conditions, SLE, SDS, PPC and POTS; however, the results from these papers are not reliable due to sample bias and hence warrant more research to understand the relationship between fatigue and HRV in patients with these diseases. In addition, the mean age of participants investigated was 43.2; therefore, it is unclear how the relationship between fatigue and HRV could be affected in clinical populations that investigate children, adolescents or young adults.

Additional confounding factors that can impact HRV include sex, medication, assessment time of day, disease stage, disease type and treatment received. Though most of the studies accounted for these types of factors, the generalisation of results could be invalid as each medical field assessed patients with different types and stages of disease. However, some methodologies excluded the eligibility of individuals on certain types of medication known to impact HRV (e.g., beta-blockers) and those with a history of cardiovascular disease and cardiac arrythmias or psychiatric disorders.

The articles included predominately measured HRV by ECG. Though ECG is deemed the gold standard, it is costly and can, therefore, be an impractical measure. Additionally, ECG monitoring can be restrictive as it requires trained technicians to perform the procedure and interpret the results and confines the individual to a controlled clinical setting that is only capable of recording heart rate for a short period [54]. Furthermore, HRV values can be affected by the length of monitoring and the ECG recording lengths varied with the shortest recording lasting 2 min, and the longest 15 min. Indeed, alongside recording time, the participants position during HRV monitoring and the device used to record the metrics were heterogenous. There is no standardised method for HRV analysis. The recent literature has discussed how the position during HRV recording can cause variation in the indices being measured [65]. Additionally, recording length has also been found to impact the reliability between HRV parameters [19]. Therefore, comparison of results warrants caution. Three of the studies measured HRV ambulatory [27,29,30], whereas seven of the studies conducted recordings whilst participants were in a supine position; however, monitoring time varied between 2 and 15 min [25,26,31,32,40,41], and one of these studies performed one of three segments for 3 min whilst participants were standing [31]. Three studies stated participants were seated with recording times between 2 and 5 min [33,34,36]. Two studies stated HRV was recorded during a vigilance test [38,39], and one study stated HRV was recorded for 8 min but the participants position was unknown [35]. Other diverse factors include the sampling frequency as well as the procedures to remove of artifacts and analyse the indices logged. Currently, there is no ‘gold-standard’ method to access HRV within clinical practice; therefore, interpretation of results should be considered with caution.

Another methodological limitation of this review is that only articles that analysed HRV using linear methods were included. Time-domain measures have been deemed simple to implement and are commonly used in practice. Frequency domains have also been accepted with research to support implantation for clinical use. Though non-linear methods are accepted in medical fields, they can be complex to implement in medical context [19,20]. Still, future studies could include non-linear parameters to provide a valuable insight into the ANS functioning.

The subjective measures of fatigue varied across each of the studies. The most used scale was the Multidimensional Fatigue Inventory Scale, which was used in four of the studies, though two of these studies used the short version. Moreover, the fatigue scales had varied characteristics and overall were inconsistent regarding the fatigue domains that were evaluated, which can impact the content and construct validity. Unfortunately, there is no universal scale that is used as a gold standard to assess fatigue in medical populations, and this could partially explain why multiple dimensions of fatigue were impacted incongruously by HRV indices. In addition, fatigue remains to be underreported and undertreated, which is due to the complexity and multifactorial nature of this phenomenon. However, all the scales that were used in these articles have all been deemed reliable and valid when assessing the targeted medical cohorts (Cronbach’s α ≥ 0.80).

The ecological validity of the articles included may have affected results as fourteen of the studies assessed HRV in laboratory conditions, whereas three studies measured HRV remotely. Therefore, factors such as living conditions, sleep habits or behaviour could have affected the parameters of HRV as well as experience of fatigue amongst these cohorts. There were three studies that involved a physically active intervention which can also affect the overall validity of findings. There was only one study in this review that collected longitudinal data for fatigue and HRV from patients with CFS to determine the relationship these variables could have overtime. Thus, it remains unclear how fatigue could be associated with HRV in the long term in different clinical cohorts. Finally, no effect size was established quantitatively (i.e., a meta-analysis), which impacts the inference of this review. Despite the discrepancies, most articles found that fatigue was associated with reduced vagal tone and overactivity of the sympathetic system, which suggests that ANS dysfunction can increase perceived fatigue in clinical populations. Moreover, the systematically reviewed literature in this paper also provides evidence that HRV could be used as an objective physiological biomarker to monitor fatigue in clinical practice.

### 4.2. Future Direction

The results from this review support an association between increased fatigue and lower HRV in clinical populations. Consequently, the findings from the reviewed articles provide evidence that HRV can be used as an objective measure to monitor fatigue in a clinical population. There were only three studies that used ambulatory methods to assess HRV. The popularity of monitoring and evaluating patients’ health remotely is becoming increasingly popular [29]. Future research could use digital technology, such as wearable devices, to monitor HRV in medical conditions, particularly those that have found fatigue to be predominant. Measuring HRV as a biomarker for fatigue could assist clinical practitioners to monitor the prevalence of fatigue. Understanding fatigue and HRV could allow for better prognosis as patients at a higher risk of experiencing fatigue could be identified at earlier disease stages if HRV is a measure to be initially assessed. Moreover, for the purpose of survival-related care, health practitioners could select interventions, such as introducing appropriate physical activity, that can improve HRV and could, therefore, mitigate fatigue to support optimising patient outcomes and improving quality of life. In summary, there is an opportunity for researchers to conduct longitudinal studies that use HRV as a physiological marker for fatigue to further strengthen the support in understanding the relationship between ANS dysfunction and fatigue across multiple medical cohorts. Using HRV as a tool for clinical assessment can assist in diagnostic evaluation and prognostic prediction to improve patient outcomes.

## 5. Conclusions

In conclusion, this systematic review of 17 articles found a significant association between fatigue and HRV for different clinical populations in 14 of the publications. The findings suggest that fatigue and HRV are related and that reduced HRV is associated with an increase in subjective fatigue. A reduction in parasympathetic activity, alongside an increase in sympathetic tone, were linked to physical, mental, cognitive and general fatigue. Therefore, HRV could be used by clinical professionals as a physiological biomarker to monitor fatigue. The monitoring of HRV can be conducted in a non-invasive manner, and recordings could be taken in either a clinical environment or ambulatory. Due to the heterogeneity of results from the articles reviewed in this paper, further investigation is warranted to deepen the understanding between fatigue and HRV. Research could evaluate the relationship between fatigue and HRV in real-world settings and assess patients from medical conditions that report a high prevalence of fatigue throughout the disease trajectory. In addition, exploring if an association between fatigue and HRV exists in younger patients could also be justified. By further understanding the relationship between fatigue and HRV, clinical professionals could use data to manage fatigue in patients to improve outcomes and quality of life.

## Figures and Tables

**Table 1:** Article Characteristics (*n* = 17).

Author and Year	Sample Size	Mean Age and Sex	Study Design	HRV Assessment	Fatigue Assessment	Main Results
Boissoneault et al. 2019 [25]	CRF = 14 HC = 14	CRF = 48.57 ± 12.11 100% women HC = 49.57 ± 13.16 100% women	Cross-Sectional Clinical Trial	ECG	VAS 0-100	Significant negative association between HRV TP and fatigue score (r = −0.70, *p* = 0.02). No other significant associations found between fatigue and LF, HF or VHF (*p* > 0.05). HC fatigue and HRV not investigated.
Capdevila et al. 2021 [26]	CFS = 32 HC = 19	CFS = 47.38 ± 1.52 100% men HC = 47.32 ± 1.51 100% men	Cross-Sectional Cohort Study	Chest Strap Polar Band H7 and FitLab app	Fatigue Index Scale-40 (FIS-40)	Significant, negative association between physical fatigue score and pNN50 (r = −0.279, *p* = 0.049) *n* = 51. No other significant results found (*p* > 0.05). Simple linear regression analysis found significant relationship between physical fatigue score and HRV parameters SDNN (β = 0.487, *p* = 0.0047), RMSSD (β = −0.394, *p* = 0.0258), LF (β = −0.537, *p* = 0.0015), HF (β = −0.421, *p* = 0.0165) and pNN50 (β = −0.378, *p* = 0.033) in CFS patients (*n* = 32). No significant results found for HC (*n* = 19) (*p* > 0.05).
Deuring et al. 2017 [27]	Allogeneic Hematopoietic Stem Cell Transplantation Survivors = 104 HC = 45	Survivors = 45.3 (median) 36.5% women HC = 41.4 (median) 44.4% women	Cross-Sectional Control Study	Vivo Metrics LifeShirt System	Functional Assessment of Chronic Illness-Fatigue (FACI-F)	GLM analysis found significant association between RSA and the fatigue scores of patients and controls (f = 6.57, *p* < 0.001). When controlling for V02 Max, a significant association between RSA and fatigue score of all patients and controls was still observed (*p* = 0.02). Contrast analysis revealed a significant association between HFP vs. NFP (t = −2.39, *p* = 0.02, d = 0.72). There was no significant association between MFP vs. NFP (t = −1.78, *p* = 0.08, d = 0.56) or NFP vs. CTL (t = 1.11, *p* = 0.27, d = 0.29). When controlling for V02 Max, a significant relationship was only detected in HFP vs. NFP (t = −2.74, *p* = 0.007, d = 0.72).
Escorihuela et al. 2020 [28]	CFS = 45 HC = 25	CFS = 46.41 ± 0.84 100% women HC = 44.96 ± 1.30 100% women	Cross-Sectional Case Control Cohort Study	Chest Strap Polar Band H7 and FitLab app	Fatigue Index Scale-40 (FIS-40)	Significant relationship discovered between all fatigue scores and all the time domain parameters of HRV (*correlation indices all* < −0.423, all *p* < 0.05) for all participants (*n* = 70). Significant relationship between all fatigue score and some frequency domains; HF (all r < −0.451), LF–HF (all r < 0.360) and HFnu (all r < −0.476), all *p* < 0.05 (*n* = 70). LF only significant for physical fatigue score (r = 0.326) and cognitive fatigue score (r = 0.322), (*p* < 0.05). Simple linear regression analysis found significant association between total FIS-40 score and mean RR (r = −0.056, *p* = 0.005), RMSSD (r = −0.055, *p* = 0.0286) and HFnu (r = −0.365, *p* = 0.0067). No significant association found between fatigue and HRV for HC.
Gashi et al. 2024 [29]	MS = 55 HC = 24	MS = 36.8 ± 9.5 64% women HC = 33.5 ± 10.6 54% women	Observational Study	Biovotion Eversion Armband (PPG Wearable Device)	Fatigue Scale for Motor and Cognitive Functions (FSMC)	HRV parameters correlated with FSMC in MS. pNN50 r = −0.40, *p* = 0.001. NN50 r = −0.41, *p* = 0.01. SD2 r = −0.47, *p* = 0.001. LF r = −0.26, *p* = 0.001. LF–HF r = 0.26, *p* = 0.001. RMSSD, SD1, HF was not significant. No fatigue and HRV were tested for HC.
Kitselaar et al. 2022 [30]	Brain Tumour Patients = 52	52.1 ± 15 44% women	Cross-Sectional Study	24 h-Holter Monitor	Multidimensional Fatigue Inventory	No significant associations reported (r ≤ 0.16, *p* ≥ 0.25).
Kristiansen et al. 2019 [31]	Patients with Acute Epstein–Barr virus (EBV) = 195 Fatigued (EBV CF+) = 91 and non-fatigued (EBV NCF−) = 104) HC = 70	CF = 17.4 ± 1.5 73.6% women NCF = 17.4 ± 1.7 57.7% women HC = 17 ± 1.8 62.9% women	Prospective Cohort Study 21-Month Follow Up	ECG (Task Force Monitor)	Chalder Fatigue Questionnaire	Weak negative correlation between LF–HF ratio response to controlled breathing and fatigue scores (T = 0.21, *p* = 0.005) in EBV CF+ group only (*n* = 91).
Martinez-Rosales et al. 2020 [32]	Systemic Lupus Erythematosus patients = 55	43.5 ± 14 100% women	Cross-Sectional Intervention Study	Polar V800	Multidimensional Fatigue Inventory	Positive correlation between LF–HF and the physical domain fatigue scores (r = 0.30, *p* < 0.05). No other significant relationships discovered.
Oka et al. 2019 [33]	15 CFS 15 CFS Control	CFS = 38.0 ± 11.1 80% women CFS Control = 39.1± 14.2 80% women	Pilot Study	ECG	Chalder Fatigue Scale	Yoga group fatigue scores and HRV (*n* = 15). Positive correlation between HF and fatigue scores (r = 0.705, *p* = 0.042). No other significant correlations detected.
Olivera-Toro et al. 2019 [34]	Spleen-Qi Deficiency Syndrome (SDS) = 67 HC = 37	SDS = 56.2 ± 4.3 56.72% women HC = 52.5 ± 6.2 54.05% women	Cross-Sectional Study	ECG	Fatigue Impact Scale Spanish Version	Linear, positive correlation between HRV and fatigue in SDS patients (r = 0.48, *p* < 0.05) (*n* = 67). HF and fatigue score negative correlation (r = −0.37, *p* ≤ 0.01) (*n* = 67).
Park et al. 2019 [35]	CRF = 25 CFS = 20	CRF = 55.52 ± 9.39 64% women CFS = 50.05 ± 9.25 65% women	Comparative Study	ECG	Fatigue Severity Scale (FSS)	CFS = FSS score significant correlation to RSSMD (r = −0.509, b = 0.032, *p* = 0.026), LF power (r = 0.484, b = 0.047, *p* = 0.036) and HF power (r = 0.508, b = 0.051, *p* = 0.026). No significant associations between FSS and pNN50, SDNN, LFnu, HFnu or HRV index. No significant associations discovered between FSS score and any of the HRV parameters CRF group.
Ryabkova et al. 2024 [36]	34 CFS 29 post-COVID 19 Condition (PCC) 32 HC	CFS = 35.0 76% women PCC = 35.0 79% women HC = 34.50 69% women	Observational	ECG	Multidimensional Fatigue Inventory	No significant correlations discovered between fatigue scores and HRV parameters (*p* > 0.01).
Rzepinski et al. 2022 [37]	MS = 53 HC = 30	MS = 45.8 ± 10.9 81.13% women HC = 40.8 ± 11.6 70% women	Cross-Sectional Study	ECG Task Force Monitor	Chalder Fatigue Scale (CFS)	MS = significant association between LF–HF ratio and physical fatigue score of CFS scale (β = −0.338, SE = 0.05, t = −2.672, *p* = 0.010). No analysis was conducted for fatigue and HRV in HC.
Sander et al. 2019 [38]	MS = 53	50.1 ± 8.7 79.2% women	Clinical Trial	NeXus-4- Biofeedback-System Blood Volume Pulse Sensor	Trait Fatigue = Fatigue Severity Scale (FSS) Tot Fatigue = VAS Trait Fatigue = Fatigue Scale for Motor and Cognitive Function (FSMC)	VLF and HF were significant predictors FSMC cognitive fatigue score (R^2^ = 0.218, f = 4.558, *p* = 0.007). No significant prediction for motor fatigue on FSMC and FSS. SDNN and pNN50 were significant predictors for tot fatigue (R^2^ = 0.241, f = 7.925, *p* ≤ 0.001). No correlation between tot fatigue and the cognitive scale of FSMC. No other significant correlations for fatigue scores found.
Sander et al. 2022 [39]	MS = 50 PMR Group = 24 SAT Group = 26	49.9 ± 8.0 80% women	Randomised Control Trial	NeXus-4- Biofeedback-System Blood Volume Pulse Sensor	Trait Fatigue = Fatigue Scale for Motor and Cognitive Function Trait Fatigue = Fatigue Severity Scale State fatigue = VAS	Mean and SD patients with weak–moderate fatigue vs. severe fatigue in SAT group pNN50 before (13.8 ± 20.1 vs. 6.0 ± 8.9) and after (12.5 ± 13.5 vs. 8.1 ± 10.1) and SDNN before (54.3 ± 33.9 vs. 36.3 ± 12.0) and after (55.4 ± 26.4 vs. 41.1 ± 14.2). Mean and SD patients with weak–moderate fatigue vs. severe fatigue in PMR group pNN50 before (8.8 ± 9.8 vs. 8.5 ± 16.4) and after (21.6 ± 18.4 vs. 10.1 ± 11.1) and SDNN before (50.5 ± 17.2 vs. 43.5 ± 28.2) and after (71.9 ± 33.7 vs. 47.7 ± 15.7). Dependent *t*-tests from post hoc testing found the PMR group with weak–moderate fatigue had a significant increase in SDNN (*p* = 0.001) and pNN50 (*p* = 0.008). No other significant differences were found.
Wheeler et al. 2022 [40]	Orthostatic Intolerance patients = 108	NHR = 37.6 ± 16.9 77% women MHR = 27.9 ± 16.0 87% women EHR = 26.6 ± 14.0 84% women	Retrospective Study	ECG	Fatigue Severity Scale (FSS)	Multiple linear regression model discovered an association between low LF power and high FSS scores (t = 2.719, *p* = 0.008). No significant relationship reported between fatigue and RMSSD or HF power (*p* > 0.05).
Zhou et al. 2018 [41]	CFS = 114 57 = Tai Chi 57 = Control	Tai Chi = <30 years = 13 30–50 years = 34 >50 years = 10 33.33% women Control = <30 years = 7 30–50 years = 37 >50 years = 13 21.05% women	Randomised Control Trial	ECG	Multidimensional Fatigue Symptom Inventory Short Form	Linear regression model revealed a significant correlation between LF–HF ratio and CRF pre (r = 0.767, *p* ≤ 0.01) and post chemotherapy (r = 0.761, *p* ≤ 0.01). No significant relationship was documented between fatigue scores and nLF or nHF (*p* > 0.05).

Abbreviations: CFS = Chronic Fatigue Syndrome, CRF = Cancer-Related Fatigue, CTL = Controls, ECG = Electrocardiogram, HER = Excessive Heart Rate Response, GLM = General Linear Model, HFP = High-Fatigued Patients, HC = Healthy Controls, HRV = Heart Rate Variability, HF = High Frequency, HFnu = High Frequency normalised, GML = General Linear Model, LF = Low Frequency, LFnu = Low Frequency normalised, LF–HF = Low Frequency–High Frequency Ratio, MHR = Moderate Heart Rate Response, MS = Multiple Sclerosis, NFP = Non-Fatigued Patients, nHF = normalised High Frequency, NHR = Normal Heart Rate Response, NN50 = mean number of times NN interval changes are greater than 50 ms, nLF = normalised Low Frequency, nLF–HF = normalised Low Frequency–High Frequency Ratio, MFP = Medium-Fatigued Patients, PPG = Photoplethysmography, pNN50 = percentage difference of NN intervals longer 50 ms, PRM = Progressive Muscle Relaxation, RMSSD = Square root of the mean sum of different squares of NN intervals, RSA = Respiratory Sinus Arrythmia, SAT = Self-Alert Training, SD = Standard Deviation, SD1 = short-term variability, SD2 = Long-term variability, SDNN = average NN intervals, TOT = Time on Task, TP = Total Power, ULF = Ultra Low Frequency, and VFF = Very Low Frequency.
